# Data-driven clinical decision processes: it’s time

**DOI:** 10.1186/s12967-019-1795-5

**Published:** 2019-02-12

**Authors:** Enrico Capobianco

**Affiliations:** Center for Computational Science, University of Miami, Miami, FL USA

**Keywords:** Big Data, Clinical decision support systems, Translational Medicine

## Abstract

Changes and transformations enabled by Big Data have direct effects on Translational Medicine. At one end, superior precision is expected from a more data-intensive and individualized medicine, thus accelerating scientific discovery and innovation (in diagnosis, therapy, disease management etc.). At the other end, the scientific method needs to adapt to the increased diversity that data present, and this can be beneficial because potentially revealing greater details of how a disease manifests and progresses. Patient-focused health data provides augmented complexity too, far beyond the simple need of testing hypotheses or validating models. Clinical decision support systems (CDSS) will increasingly deal with such complexity by developing efficient high-performance algorithms and creating a next generation of inferential tools for clinical use. Additionally, new protocols for sharing digital information and effectively integrating patients data will need to be CDSS-embedded features in view of suitable data harmonization aimed at improved diagnosis, therapy assessment and prevention.

In this Editorial we announce a new section in the *Journal of Translational Medicine*.

A data-driven clinical decision support system (CDSS) is commonly conceived as a tool for (a) Managing complex tasks, such as combining a chronologically ordered variety of evidenced conditions, symptoms, tests and other data types all available to the clinician, and (b) Delivering snapshots of the patient’s health status either at a given time or along a temporal trajectory. The tools necessarily embed heterogeneous knowledge bases (of genetic, omic, epigenomic, exposomic nature etc.) used to cross-reference the patient’s characteristics by the means of algorithms.

The goal is to optimize recommendations and decisions to the benefit of the patient. To such purpose, screening time, extension and frequency of measurements should be suitably dilated while preserving accuracy and confidence. Despite limitations remain, say non-inclusion of certain risk factors, lack of rare evidences, insufficient variety of data types etc., the advantages are multiple for the individual patient, especially in terms of intervention, and in particular to correct non-administered, ineffective, wrong or even unneeded treatments. When the reference is a patient’s group, the expected advantages assign centrality to cost-effectiveness data. The data must be reported accurately to reflect health providers’ performance and leverage consistent study design and assessment of patient outcomes.

Systematic CDSS reviews have appeared (see for instance [1]). CDSS are tools designed to enhance patient care by consolidating patient profiles via repeated merging of clinical knowledge and patient information [2]. This therefore is a process whose multiple observations or measurements or assessments cover landmark points such as first consultation, then diagnosis, follow up, hospitalization etc. [3]. Past literature has associated CDSS to Electronic Health Records (EHR) [4] due to the presence of clinical guidelines, alerts, reminders and similar. However, a static view of a dynamic process is by definition just an approximation without specifying the mechanisms governing the dynamics.

Criticisms have also appeared [5, 6], sustaining that the majority of studies have failed to demonstrate outcome improvements or highly significant results. The clear indication is that in order to become components routinely adopted in clinical practice, CDSS should be further developed for the sentiment around them being also revised. To enable such changes, CDSS processed information must leverage increasingly connected data evidenced in disparate but integrable sources and digitalized environments (a starting point is offered by *Informatics for Integrating Biology and the Bedside* (http://www.i2b2.org), one of 7 *NIH Roadmap for Biomedical Computing* (http://www.ncbcs.org) funded centers, and related parallel initiatives).

Some of the challenges ahead involve CDSS knowledge bases, and posit questions such as:

i. How to improve widespread access to biomedical data?
ii. How to lower barriers to the novel clinical trials development and patient health records?
iii. How to transfer Big Data knowledge into point-of-care systems?
iv. How to exploit Patient-Reported Outcome Measures in real time care through patient engagement etc?

The relevance of these questions for Translational Medicine is evident, as wells as it is necessary to shape the ability to combine data-driven evidences, identify signals and find patterns that address questions from which to test new hypotheses specific to the patient, and finally define risk scenarios involving groups of patients sharing features that once reconciled with new data types may drive effective prevention and/or more timely targeted therapies.

Other CDSS challenges [7, 8] are methodological, say

- How to ensure accurate probability estimations for risk assessment, diagnosis, therapeutic intervention and prognosis?
- How to optimize data calibration with discrimination in predictive learning models?
- What is the best possible complementary evidence in support to validation for scopes of prognostic use of CDSS?

Other challenges are of an ethical dimension [9, 10], in view of:

- What hierarchy of evidence can guide clinical decision making at reduced risk of bias?
- Should expensive therapies be considered when facing marginal survival chances or to what extent an algorithm can influence decisions on probabilistically determined critical conditions?
- How to responsibly prioritize secondary findings in the context of treatment?

This new section of the *Journal of Translational Medicine* aims to attract multidisciplinary research and promote scientific interactions, and expects to accelerate the establishment of CDSS in the clinical practice.

## References

[1] Melton BL (2017). Systematic review of medical informatics-supported medication decision making. Biomed Inform Ins..

[2] Osheroff JA, Teich JM, Middleton B, Steen EB, Wright A, Detmer DE (2007). A roadmap for national action on clinical decision support. J Am Med Inform Assoc.

[3] Castaneda C, Nalley K, Mannion C, Bhattacharyya P, Blake P, Pecora A, Goy A, Suh KS (2015). Clinical decision support systems for improving diagnostic accuracy and achieving precision medicine. J Clin Bioinform..

[4] Jha AK, DesRoches CM, Campbell EG, Donelan K, Rao SR, Ferris TG, Shields A, Rosenbaum S, Blumenthal D (2009). Use of Electronic Health Records in U.S. Hospitals. N Engl J Med.

[5] Jaspers MW, Smeulers M, Vermeulen H, Peute LW (2011). Effects of clinical decision-support systems on practitioner performance and patient outcomes: a synthesis of high-quality systematic review findings. J Am Med Inform Assoc.

[6] Bright TJ, Wong A, Dhurjati R, Bristow E, Bastian L, Coeytaux RR, Samsa G, Hasselblad V, Williams JW, Musty MD, Wing L, Kendrick AS, Sanders GD, Lobach D (2012). Effect of clinical decision-support systems: a systematic review. Ann Intern Med.

[7] Jiang X, Osl M, Kim J, Ohno-Machado L (2012). Calibrating predictive model estimates to support personalized medicine. J Am Med Inform Assoc.

[8] Jiang X, Boxwala AA, El-Kareh R, Kim J, Ohno-Machado L (2012). A patient-driven adaptive prediction technique to improve personalized risk estimation for clinical decision support. J Am Med Inform Assoc.

[9] Chow N, Gallo L, Busse JW (2018). Evidence-based medicine and precision medicine: complementary approaches to clinical decision-making. Prec Clin Med..

[10] Fischer T, Brothers KB, Erdmann P, Langanke M (2016). Clinical decision-making and secondary findings in systems medicine. BMC Med Ethics..

